# Oocyte Aging: A Multifactorial Phenomenon in A Unique Cell

**DOI:** 10.14336/AD.2023.0527

**Published:** 2024-02-01

**Authors:** Pawel Kordowitzki, Szymon Graczyk, Amin Haghani, Michael Klutstein

**Affiliations:** ^1^Department of Preclinical and Basic Sciences, Faculty of Biological and Veterinary Sciences, Nicolaus Copernicus University, Torun, Poland.; ^2^Department of Human Genetics, David Geffen School of Medicine, University of California Los Angeles, Los Angeles, CA, USA.; ^3^Altos Labs, San Diego, CA, USA.; ^4^Institute of Biomedical and Oral Research, Hebrew University of Jerusalem, Jerusalem, Israel

**Keywords:** methylome, DNA-methylation, epigenetics, heterochromatin, oocyte, egg, aging, ER stress, protein folding, fibrosis, Stella, TET3, UHRF1

## Abstract

The oocyte is considered to be the largest cell in mammalian species. Women hoping to become pregnant face a ticking biological clock. This is becoming increasingly challenging as an increase in life expectancy is accompanied by the tendency to conceive at older ages. With advancing maternal age, the fertilized egg will exhibit lower quality and developmental competence, which contributes to increased chances of miscarriage due to several causes such as aneuploidy, oxidative stress, epigenetics, or metabolic disorders. In particular, heterochromatin in oocytes and with it, the DNA methylation landscape undergoes changes. Further, obesity is a well-known and ever-increasing global problem as it is associated with several metabolic disorders. More importantly, both obesity and aging negatively affect female reproduction. However, among women, there is immense variability in age-related decline of oocytes’ quantity, developmental competence, or quality. Herein, the relevance of obesity and DNA-methylation will be discussed as these aspects have a tremendous effect on female fertility, and it is a topic of continuous and widespread interest that has yet to be fully addressed for the mammalian oocyte.

## 1. Introduction

**Figure 1.**
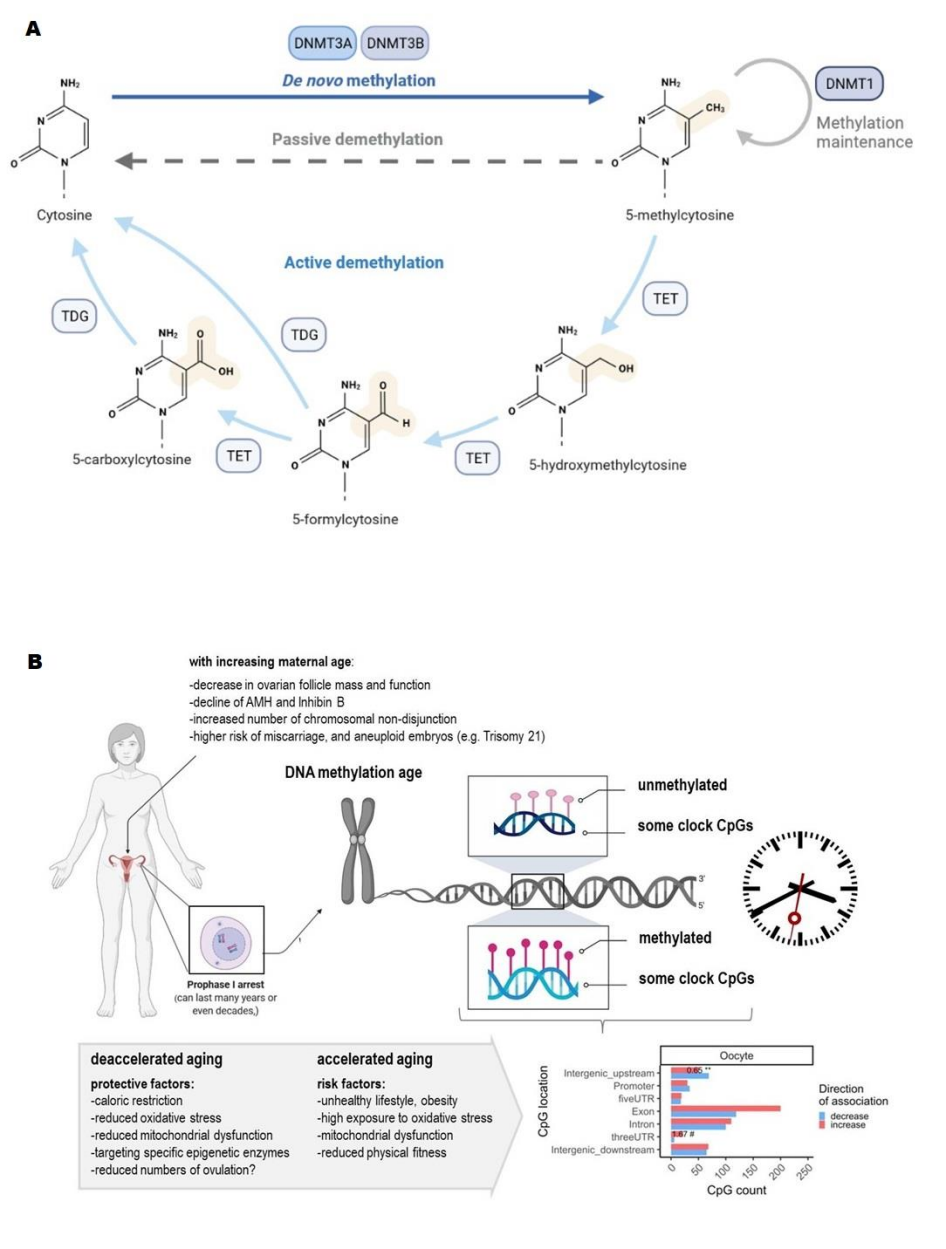
**DNA methylation in human eggs with regards to aging**. (**A**) Scheme showing the mechanism of methylation and demethylation of cytosine. (**B**) Scheme showing the consequences of advanced maternal age (especially in women at the age of 35 years and above) and how an accelerated and a deaccelerated aging process could impact the DNA-methylation age of oocytes. The oocytes in ovarian follicles are arrested in Prophase of the first meiotic division, and with advancing maternal age, more errors are likely after the resumption of meiosis what could lead to aneuploidies in the offspring (e.g., Trisomy 21/Down-Syndrome). *Abbreviations:* AMH=Anti-Muellerian Hormone, DNMT= DNA methyltransferase, TDG= Thymine DNA glycosylase, TET= ten-eleven translocation.

The oocyte is considered to be the largest cell in mammalian species, and very unique with regard to its lifespan since aging-related changes in ovaries are detectable very early in life [1]. Women are born with a large pool of oocytes in primordial follicles, arrested in prophase of the first meiotic division (Fig.1). Because oocytes are metabolically active, they experience the effects of chronic exposure to environmental influences. Due to attrition, ovulation, and apoptosis, the number of eggs decreases continuously throughout life, to approximately 300,000 at menarche. This number drops to nearly complete exhaustion by menopause. With important implications, women around the world increasingly postpone childbearing, leading to reduced fertility, more abortions, fetal abnormalities, and fewer children per family on average [2,3]. In parallel, the utilization of in vitro fertilization (IVF) has increased, along with a significant economic burden on society. There is an obvious lack of research on how to improve fertility in older women and most IVF centers routinely refer women to third-party egg donation after ages 42-43 years [4]. Therefore, novel biomarkers of ovarian aging and new intervention techniques, would be desirable and epigenetics could play a significant role in this process. Despite rapidly improving our knowledge about the aging methylome, we have yet to fully understand the underlying mechanisms that underpin age-related epigenetic alterations that constitute one of the primary hallmarks of aging [5]. The methylation of cytosines (Fig. 1) has, for example, been well-investigated [6,7]. 5-methyl cytosine (5mC) on promoters of genes almost invariably relate to transcriptional repression, in concert with other transcriptional silencing mechanisms such as the histone modifications. DNA methylation (DNAm) contributes to a multitude of biological processes, including regulation of gene expression, X-chromosome inactivation, and aging [7]. As the methylome acts as one of the important interfaces between the genome and the environment, it also responds to environmental stimuli from lifestyle factors such as diet and smoking [4].

**Figure 2.**
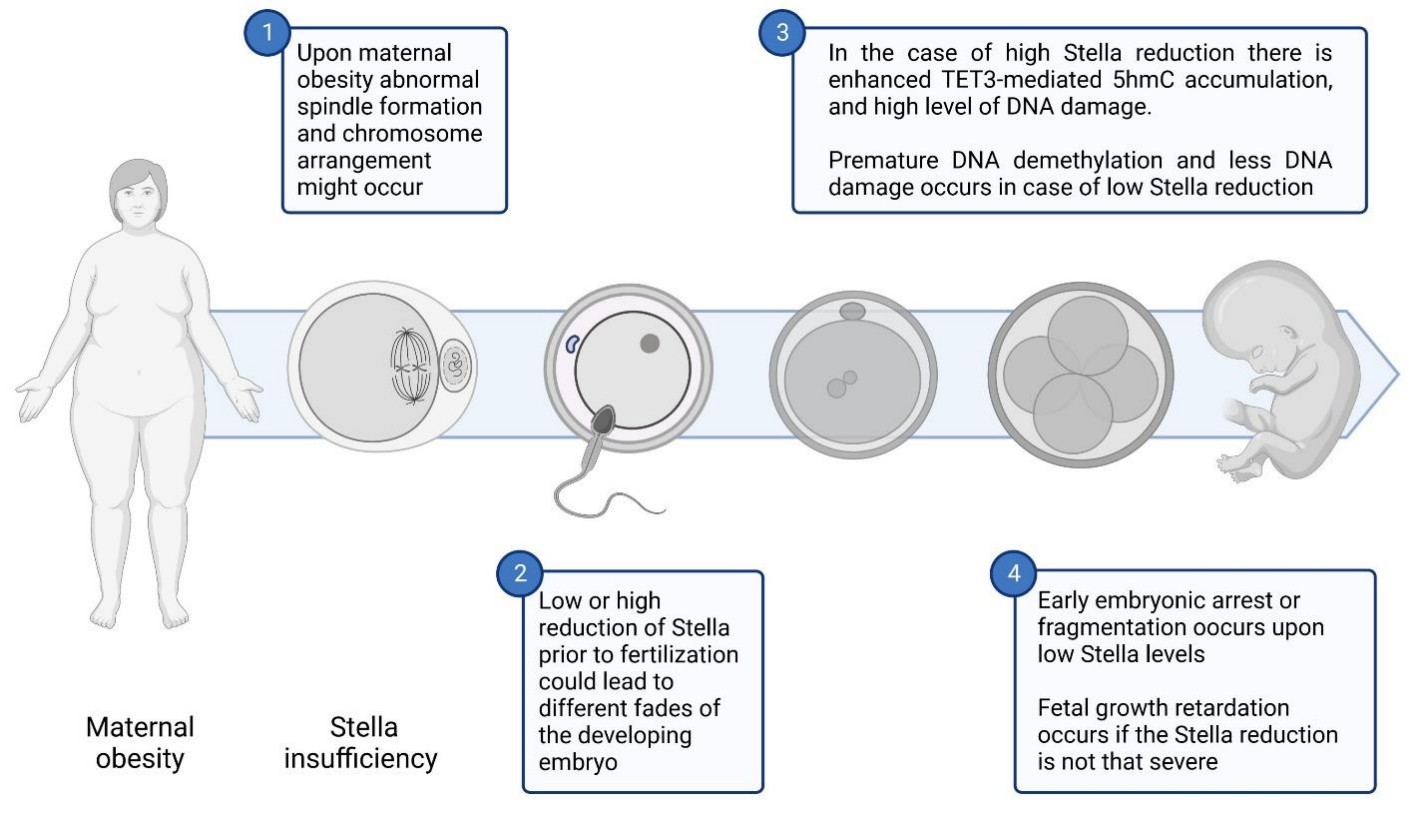
Scheme showing potential outcomes in case of maternal obesity and Stella insufficiency.

The recent development of novel DNA methylation microarrays enabled the investigation of DNA methylation in different mammalian species and cell types and has opened the possibility of developing third generation (multi-species) epigenetic clocks, which can serve as powerful tools to measure biological aging [6,7]. These methylation arrays can also be used for numerous purposes in reproductive medicine to advance the field and open new possibilities. Furthermore, obesity has been reported to be a risk factor for developing deficiencies [8,9]. Decreased embryo quality and impaired folliculo- and steroidogenesis are the main changes that occur in the female reproductive system as a result of obesity [10,11]. There is no doubt that due to ethical and legal issues, and limited availability, research on human oocytes is restricted in numerous countries. Therefore, quite often an adequate animal model is needed. Both bovine and murine oocytes are an attractive animal model but the degree of similarity to human oocytes differs. For instance, the murine IVF system and embryo culture are frequently taken into consideration as a model for human IVF [12]. With regard to DNA methylation, it is widely accepted that the oocyte undergoes re- and de-methylation during oocyte development. Noteworthy, there are two waves of genome-wide reprogramming in mammals, namely in germ cells and in preimplantation embryos. Interestingly, the step of epigenetic reprogramming in germline cells, including the egg cell, is essential for the phenomenon of imprinting [13]. Noteworthy, the big hyper- and hypomethylated domains which are associated with transcription appear to be similar in human and murine eggs. However, there is a significant difference with regard to the methylated regions in human and murine egg cells, reflecting contrary transcriptome profiles. In addition, the maternal genome appears to be demethylated at a lower level in human blastocysts when compared to murine counterparts (Fig. 2), and the active DNA demethylation by TET enzymes is conserved but is less pronounced in human zygotes [14]. Furthermore, it is well known that there are specific regulators which control global DNA methylation, histone modification, transposon elements, and the energy metabolism of the oocyte [15-18].

Therefore, in this Review, we aim to provide a summary of recent research related to maternal aging, obesity, and DNA methylation in regard to their impact on the mammalian oocyte - the crucial female cell for procreation.

## 2. Epigenetic alterations as a hallmark of oocyte development and aging

The epigenome and specifically, the DNA methylome undergoes very significant changes with oocyte growth and development. DNA of primordial germ cells is highly methylated. When these cells enter the genital ridge, global DNA methylation decreases drastically, except for primary imprinted regions, which escape this process, and retain their DNA methylation state. During gametogenesis, at the time when oocytes are formed, de novo methylation of DNA occurs gradually with oocyte growth. This genome-wide demethylation and re-methylation are defined as the first round of epigenetic reprogramming. The second round of such reprogramming occurs in the embryo after fertilization. DNA demethylation after fertilization occurs both actively and passively. Active demethylation is promoted by the ten-eleven translocation (TET) enzymes (Fig. 1) which catalyze the conversion of 5MC to 5-hydroxmethylcytosine (5hMC). From the very early stage of development, methylation and demethylation of the DNA occur in very specific manner and locations. Depending on the specific chromatin context, some genomic loci gain heterochromatin and DNA methylation, and some heterochromatic regions lose their repressive marks with resulting de-repression of the locus [19]. For example, CpGs located in polycomb repressor type 2 complex targets tend to gain methylation with age, - particularly in peripheral tissues [6]. Strikingly, a recent study revealed that oocytes do not follow this pattern [20] suggesting a distinct epigenetic aging pattern in comparison to other organs. This may be related to the specific and specialized pattern of DNA methylation in oocytes. Interestingly, histone modifications dramatically change with age in oocytes. As demonstrated recently, mouse and human oocytes lose heterochromatin histone markers with age [19]. This loss results in a massive activation of transposable elements and retrotransposons which result in DNA damage. Consistent with this, it has been demonstrated that inhibition of reverse-transcriptase activity or artificially increasing heterochromatin levels in aging oocytes abrogates DNA damage and improves oocyte in-vitro maturation efficiency when applied to aged oocytes [19]. In addition, post-translational histone modifications (PTMs) modulate transcriptional activity [21] in the egg and, as part of their meiotic function, they are of importance for the processes of kinetochore formation and chromosome segregation [22]. Noteworthy, changes in histone PTM patterns and DNA methylation are typical for oocyte oogenesis and maturation [23], and appear to be sensitive in response to stress, stimulation, and aging [22]. A recent study provided evidence that the chromatin structure in post-pubertal murine oocytes has fewer chromocenters than prepubertal counterparts, and heterochromatin marker values, such as H3K9me2, H3K27me3, or H4K20me1 increase significantly at the onset of puberty [22]. In contrast to the latter-mentioned phenomenon, euchromatic markers behave in a different fashion, namely, H3K4me3 levels are elevated during puberty, whereas H3K27Ac shows lower values [22]. Moreover, the same study revealed that the application of follicle-stimulating hormone (FSH) to pre-pubertal oocytes for 24h impacts their chromatin structure to a post-pubertal configuration and elevates their histone methylation levels. In consequence, it appears that FSH plays a key role in chromatin regulation during the pubertal transition [22]. In addition, H3K9me3 is linked to transcriptionally silent heterochromatin, and it has been shown that KDM4A alters the entire H3K9me3 landscape in mature eggs at sites occupied by bdH3K4me3, emphasizing the crucial role of KDM4A in maintaining the integrity of the maternal epigenome, which is a prerequisite for adequate zygotic genome activation and the transfer of developmental control to the embryo [24]. It is worth mentioning that ribosomal DNA (rDNA) methylation seems to reflect functional changes in nucleolus biology during aging and aging-related pathologies [25], and aging-related rDNA methylation has been proposed as a universal predictor of age [26]. A recent study provided evidence that there is an aging-related elevation of rDNA methylation levels in murine eggs generated from advanced-age mothers [27]. In consequence, ovarian aging appears to be associated with increased values of rDNA methylation in meiotically arrested oocytes. However, eggs from the same woman can have different levels of rDNA methylation and, by extrapolation, different epigenetic age [27]. The latter-mentioned fact contributes among others to the variability of oocytes’ developmental competence even when generated from the same woman. As already mentioned in the Introduction, previous research provided evidence that transcriptional and epigenetic changes, particularly DNA methylation, appear to contribute to the deterioration in oocyte quality with regard to maternal age [28]. With advancing maternal age, there is a chronic exposure of oocytes in the ovary to a changing environment, such as altered hormone levels and changes in energy and single-carbon metabolism, which affect gene expression and epigenetic processes, and in consequence, may contribute to lower viability of oocytes [29]. The impact on the epigenetic quality of the oocyte is also important since it can potentially be inherited by the embryo, which is manifested in the later developmental outcomes of the offspring. Besides, one should keep in mind that gene expression in the oocyte is unique in that it contains many oocyte-specific transcripts that regulate pathways and functions not only in the oocyte itself but also during early embryonic development [30]. This has been emphasized in a recent study [30], in which a diminished complexity and elevated variability in the transcriptome of eggs from older women have been described. The transcriptome heterogeneity allows the division of oocytes with an immature chromatin configuration transcriptome into two groups. The first group is composed of those oocytes with reduced developmental competence, which is indicated by lower expression of maternal effect genes, and the other group contains those oocytes with a juvenile transcriptome. The same study revealed that oocytes from older women had, on average, reduced CpG methylation, but the characteristic landscape of bimodal oocyte methylation was preserved [30]. For most differentially expressed transcripts, the lack of related methylation changes suggests that most age-related effects on the transcriptome are on a post-transcriptional basis. Interestingly, the oocytes differed in the expression and methylation of genes, which may have contributed to the differential oocyte competence or penetrance of age-related maternal phenotypes in the offspring [30].

## 3. An oocyte epigenetic estimator of chronological age

Researchers have taken advantage of age-related DNA methylation changes to develop epigenetic estimators of chronological age, known as epigenetic clocks. Epigenetic age, which is the quantitative prediction of such clocks appears to capture some elements of biological age [20]. Epidemiological studies revealed that individuals with epigenetic ages that are older than their chronological ages have increased risks of various chronic diseases [31]. The application of epigenetic clocks is not limited to humans but can be extended to different mammalian species [32,33]. Recently, a universal mammalian methylation clock, based on highly conserved CpGs across mammals, was developed. This clock is able to estimate biological age of all mammalian species from DNA derived from different tissues and organs [6]. Due to ethical restrictions for research on human eggs [1], bovine eggs are a good model to facilitate future studies into the epigenetic underpinnings of human female reproductive aging. It has been observed that germinal vesicle stage oocytes exhibit a slower rate of epigenetic aging, but surprisingly, they start with an older developmental epigenetic age than a non-germline tissue such as blood [20]. This observation parallels our prior statement about a lack of methylation gain in the oocyte’s promoter regions, shedding new light on the phenomenon of oocyte aging. Evidently, oocytes are somehow protected from the gain of methylation in the regulatory regions. This observation also matches the reported oocyte protection against mitochondrial DNA point mutations and differs from aging of somatic tissues [34]. It thus appears that the epigenetic clock ticks differently in oocytes, even early in life, suggesting a rejuvenation event during early embryonic cleavage stages followed by aging, as shown in a recent study [35]. In this study, zygotes were reported to be epigenetically older than cells from blastocysts (Fig. 1). With the help of these new insights into the epigenetic age of mammalian oocytes, researchers will hopefully be able to address several open questions regarding the nature of reproductive aging in women. A blood-based oocyte epigenetic clock can be a scalable measure to examine oocyte aging and identify genetic variants or environmental factors (e.g., lifestyle and diet) contributing to healthy and extended reproductive lifespans. Reproductive aging does not only have significant relevance for women’s health but also profoundly affects society. Moreover, studies of reproductive aging may result in new knowledge about more fundamental pathways of aging involving epigenetic reprogramming, and genomic imprinting. To what extent epigenetic modifications in oocytes are driven by lifestyle changes, different environmental conditions, and the use of reproductive biotechnologies is important to understand. Interestingly, a recent mouse model provided evidence that the oocyte’s methylome is robust and is not drastically altered by artificial reproductive techniques (ART) [36]. To achieve breakthroughs, future studies of oocyte epigenetics should be directed toward epigenetic reprogramming and how oocytes are able to encode and store epigenetic information which has been set very early, even before birth.

## 4. Relevance of Stella, TET3, UHRF1 for DNA methylation and the oocyte

DNA methylation is not necessarily associated with oocyte developmental competence however, it is an essential factor for genomic imprinting [37], and disorders associated with abnormal methylation of given loci, cause disorders related to metabolism, placental and fetal growth [37]. This regulation takes place at so-called imprinting control regions, where the higher methylation of given loci, the more strongly the inhibition of a given gene is expressed. Therefore, the proper methylome status is crucial for genomic imprinting [38,39]. The DNA methyltransferase (DNMT) family, which consists of 6 pillars, is responsible for maintaining the proper methylome status of both specific imprinting control loci and other genes: DNMT1, DNMT2, DNMT3A, DNMT3B, DNMT3L, and the male-specific DNMT3C. Altogether, they are crucial for epigenetic regulation [40]. DNMT3A and DNMT3B are known to be enzymes required for de novo methylation, but their knockout does not alter already established methylation patterns. DNMTL3 is known more as a cofactor for the previous two because it lacks active catalytic sites [41], but interestingly, it has a site on its C-terminal chain for DNMT3A and DNMT3B binding increasing its ability to bind chromatin in the oocyte which significantly improves their catalytic activity [42]. In the female oocyte, the knockout of DNMT3A and DNMTL3 binding gene is associated with the loss of maternal phenotype and the failure of genomic imprinting [43]. Another enzyme responsible for de novo methylation is DNMT3C, but its action is reduced to male lineage methylation of evolutionarily young retrotransposons [44]. Moreover, mutation of this gene in mice can lead to diseases found in humans [45]. DNMT's role in epigenetic regulation is crucial for maintaining methylome balance and thus proper oocyte growth at least up to the metaphase II stage [46].

The Stella protein, also known as Dppa3 and Pgc7, is highly expressed in primordial germ cells [16,47]. Its main action is based on the specific control of global DNA demethylation enabling the epigenetic remodelling process immediately after fertilisation [48]. While demethylation in the male pronuclei is almost instantaneous, this process is delayed in the female pronuclei of the zygote [18,49]. Thus, a model of epigenetic asymmetry between the female and male pronuclei is drawn. The establishment of global hypomethylation occurs through the oxidation of 5-methylcytosine (5-mc) to 5-hydroxymethylcytosine (5-hmc) [17,50]. This reaction is tightly controlled by the Stella protein by which an extended demethylation time is possible (Fig. 2) [16]. Oocytes from mice fed on a high-fat diet (HFD) have shown a strong signal for 5-mc, while the signal for 5-hmc was markedly reduced, suggesting a severe disruption of DNA demethylation [18]. Some research reported that the conversion of 5-mc to 5-hmc is possible due to the binding of Stella to demethylated lysine 9 on histone H3 (H3K9me2) [51], which was not confirmed by other studies, indicating that H3K9me2 does not appear to be the main fraction interacting with Stella in delayed DNA demethylation. There is no doubt that adequate levels of maternal global DNA methylation are crucial for proper oocyte development and survival [52]. In early embryos, transposable elements play a particular role by dynamically re-organising the arrangement of available chromatin [53]. Given the critical role of the Stella protein for the oocyte, its deficiency in a HFD mouse model has led to hypomethylation of transposable elements [54]. Interestingly, inducing overexpression of the Stella protein may prevent the above defects to some extent by inhibiting overaccumulation of 5-hmc, but some genomic loci did not respond to the increase in Stella activity [55]. These data suggest that despite the important function of Stella in maintaining DNA methylation, there may be other factors contributing to global hypomethylation in oocytes of obese mothers.

**Figure 3.**
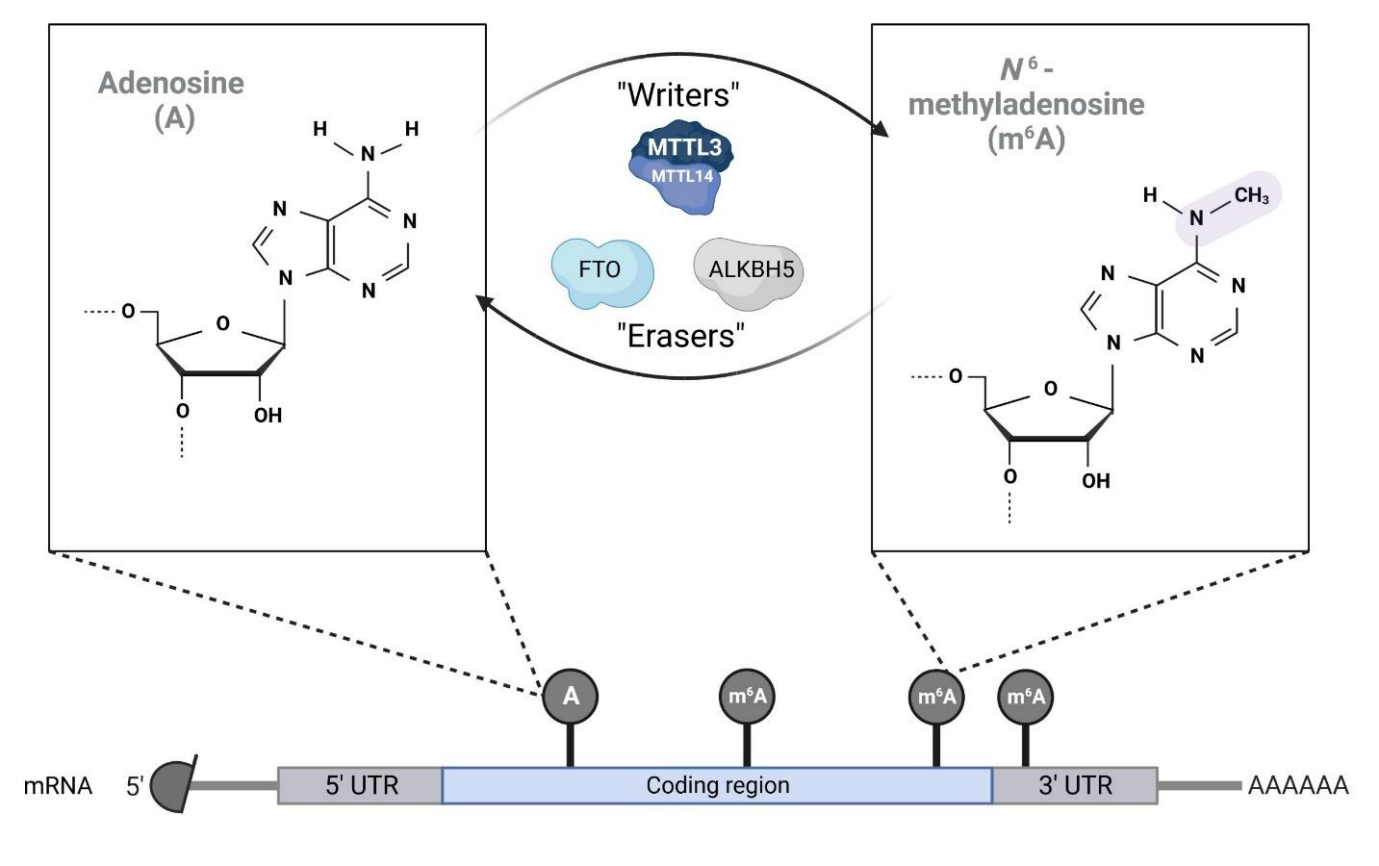
**Scheme showing the working mechanism of the so-called writers and erasers**. Writers are the MTTL3-14 complex, and the so- called erasers, are FTO and ALKBH5, which are necessary for the transfer of methyl groups between adenosine (A)and N6-methyladenosine (m6A).

The TET3 dioxygenase is considered to be one of the critical control points catalysing the conversion of 5-mc to 5-hmc only in the male pronucleus (Fig. 3) [49]. In addition to its catalytic function, TET enzymes play a key role in the expression of transposable elements as well as chimeric transcripts, both of which are important processes for the completion of zygotic genome activation (ZGA) [56]. HFD-fed mouse oocytes showed increased levels of TET3, suggesting that reduced Stella expression in HFD oocytes contributes to the activation of pathways for TET3 leading to overly rapid demethylation of the transgenome [55]. Additionally, after deletion of the gene encoding for TET3, a reduction in γ-H2AX was observed [55], which, in consequence, illustrates the extent of double-strand DNA damage in mouse oocytes [57].

Uhrf1 (ubiquitin-like, containing PHD and RING finger domains 1), also known as NP95 in mice and ICBP90 in humans, is a protein involved in DNA methylation. To maintain an appropriate level of DNA methylation, the interaction of UHRF1 protein with DNA methyltransferase 1 (DNMT1) takes place [15]. It has been shown that the Stella protein can attenuate the action of UHRF1 protein thus indicating another pathway for the regulation of global DNA methylation in mouse oocytes [47]. Recently, attempts have been made to understand the exact interaction between the Stella protein and UHRF1 protein. Interestingly, the knockout of the gene encoding for Stella resulted in a marked increase in global DNA methylation, where 22% of the CpG islands were hypermethylated, and the location of UHRF1 protein was detected almost exclusively in the nucleus [58]. Such an increase in global DNA methylation may be due to occupying binding sites of inactive regions of the transgene by DNMT1 during oocyte maturation [58,59]. In addition, there is another mechanism whereby the PHD-UHRF1 domain and the Stella protein compete for the same binding site on the Histone H3K9 tail, particularly the trimethylated one [47]. Interestingly, it has been revealed that the Stella protein through a strong binding to the PHD-UHRF1 domain, displaces the latter-mentioned complex from its binding site on H3K9me0-3, thus preventing DNA methylation [47]. Ectopic overexpression of Stella has been shown to largely restore the normal state of global DNA methylation in oocytes [58]. Stella protein deficiencies may also lead to severe consequences in the pre-implantation period (Fig. 2). Stella knockout led to a developmental interruption of two-cell embryos, and only 15% developed into blastocysts [58,60], which is consistent in another research [61]. In summary, the before-mentioned results indicate that the absence of Stella leads to an excessive DNA methylated oocyte. Knowing the importance of the interaction between the Stella protein and UHRF1 protein in future studies will be worthwhile to highlight the state of global methylation regulated by UHRF1. For instance, by using a HFD mouse model, this could further elucidate the defective mechanisms of global DNA methylation regulated by Stella in obese mothers.

## 5. N6-methyladenosine: An epitranscriptomic mRNA modification

During oocyte development, mRNA undergoes continuous epigenetic modifications which enable normal embryonic growth and development [62]. Through methylation of the relevant nucleobase pairs, tight control of gene expression is possible [62]. The most abundant modification is the methylation of the sixth nitrogen at adenosine, which is transferred from the S-adenosylmethionine moiety of the RNA chain resulting in N6-methyladenosine (m6a) [63]. This modification is enabled by a complex of methyltransferases known as 'writers', such as the METTL3/14 complex (Fig. 3) [64], and associated fractions including WTAP [65], KIA1429 [66], RBM15 [67], ZCH13 [68,69], and METTL16 [70]. Excessive methylation is associated with excessive silencing of gene expression, so demethylases termed 'erasers' remain in close correlation with the methyltransferase complex [71]. Prominent among these are the obesity-related protein (FTO) [72] and ALKBH5 (Fig. 3) [73,74]. Recently described important regulatory sites of the m6a formation, are the YTH domain-containing binding proteins [75], and the IGF2 mRNA binding protein subfamily [76]. Interestingly, abnormalities in the expression of the METTL3 gene, the so-called m6a subunit, are associated with high fetal mortality and impaired oocyte development. Inactivation of the METTL3 gene leads to early embryonic lethality, with all embryos being lost in the METTL-cKO group and 232 being born in the F1 generation of the control group [77], confirming the previous studies [78]. Interestingly, deletion of the METTL3 gene had no effect on the follicles of 6-week-old murine pups; however, a decreased number of follicles was observed when growing old [77]. METTL3 has been reported to maintain high activity in terminal follicles, thus sustaining oocyte development by stabilizing functional maternal mRNA. Previous research provided evidence that there is an essential link between Intersectin1, which has a crucial function in microtubule formation and normal meiosis [79], and IGF2BP3, a cofactor that maintains levels of Itsn1 in terminal follicles. Interestingly, as the gene for METTL3 was knocked out, impaired meiotic maturation in oocytes was present, suggesting the disruption of the before-mentioned link between Itsn1 and IGF2BP3. Similar conclusions have been drawn by Zhang et al. [80], who reported high levels of double-stranded DNA breaks after METTL3 gene knockout. Among the components of the m6a heterodimer (Figure 3), KIA1429 has been identified as a maternal gene [81]. Although its function remains elusive, previous studies have provided insight into its essential interaction in the deposition of m6a onto maternal mRNA during activation of the zygotic genome [82]. Another important regulator of proper mRNA methylation is FTO [83]. It is known to be a key control of early embryonic development and is essential for normal adipogenesis [84]. A recent study by Wei et al. [85] reported the important role of FTO in maintaining proper methylation of chromatin-associated RNA in embryonic stem cells. The induced inactivation of the FTO gene revealed significant defects in oocytes with regard to the hypermethylation of RNA transcriptomes, particularly in the long interacting element 1 (LINE1), which is an important regulator of global chromatin availability in developing early embryos [86]. Given that most mRNA modifications are regulated by m6a, this complex has attracted particular interest among researchers in recent years, since its critical role in embryonic development has been demonstrated. Previous research provided strong evidence that the m6a complex is a critical regulator of maternal mRNA expression and ovarian follicle growth [80,82]. Deletions of key factors for normal mRNA methylation caused severe mRNA methylation disruption leading to oocyte growth inhibition, impaired oocyte development and the occurrence of early embryonic lethality [87]. As obesity induces serious problems related to global DNA methylation of growing oocytes and pre-implantation embryos [18,55], future studies regarding obesity and its effects on the m6a complex may provide further insights into infertility problems in obese women.

## 6. Impact of obesity and metabolic changes on oocyte development

Nowadays, obesity among women is becoming a more and more serious problem globally [88]. It is a well-known fact that obesity significantly affects women's health leading among others to polycystic ovary syndrome, premature menopause, or impairment to maintain pregnancy [89]. Besides, it leads to in-utero abnormalities of the developing fetus, leading to macrosomia, stillbirth, or intensive care of the newborn [88]. Obesity negatively affects the microenvironment of ovarian follicles and the oocyte itself through energy deficiencies of the oocyte, or disorganization and misalignment of chromosomes [90-92]. Obesity-related mechanisms by which the oocyte developmental competence is disrupted are for instance chronic inflammation, oxidative stress, or energy imbalances. Triglyceride accumulation in adipocytes leads to their hypertrophy and consequent necrosis which stimulates macrophages and T lymphocytes to produce pro-inflammatory cytokines i.e., IL-1, IL-6, and TNF-alpha. Thus, latent chronic inflammation negatively affects all tissues of the body, including ovarian tissue and, consequently, oocytes [93]. This is confirmed by a study by Nteeb et al. [94] who postulated that mice fed a high-fat diet showed a high immune activity within the ovary which negatively affected its function. Remodeling of the intestinal microbiota leads to increased permeability for enterocytes, allowing the release of lipopolysaccharides into the bloodstream. Furthermore, the binding to toll-like receptor 4 induces a cascade leading to the activation of the pathway for NFκB phosphorylation p65 and another pathway for the synthesis of pro-inflammatory cytokines namely TNF-alpha and IL-6. In addition, this pathway leads to the activation of reactive oxygen species in the form of O2-, which are then catalyzed to hydrogen peroxide due to the action of superoxide dismutase [90]. Together, these mechanisms act in a positive feedback loop, establishing a state of chronic inflammation that does not necessarily produce clinical symptoms, but significantly affects a number of body systems, including the female reproductive system.

Energy supply and balance for proper oocyte maturation and spindle assembly are of paramount importance. During oocyte development, the number of mitochondria can reach up to 160,000 [95] due to the fact that they are essential for the formation of the karyokinetic spindle, the polarization of chromosomes and the ejection of first and second polar bodies [96]. Hence, mice fed a high-fat diet experience a number of structural changes in mitochondria manifesting as changes in energy availability and thus oocyte incompetence [97]. In addition to structural changes, abnormalities in the expression of specific proteins for the maintenance of energy homeostasis in the oocyte have also been observed. Sirtuin 3 (SIRT3) is involved in maintaining constant energy levels in maturing oocytes, thereby preventing DNA damage through ROS accumulation [98]. Superoxide dismutase 2 (SOD2) has been shown to be closely related to SIRT3, where its activation and reduction of ROS occurs through SIRT3-regulated deacetylation [99]. In HFD-fed mice, there is a decrease in SIRT3 expression, and consequently what raises SOD2K68 acetylation levels contributes to ROS accumulation in the oocyte [100]. In addition, chromosome misalignment and disorganization of the karyokinetic spindle with dysfunction of the SIRT3-SOD2 axis has been observed in HFD mice [101]. An important regulator of oocyte and non-oocyte energy metabolism is the TIGAR gene, involved in DNA repair and upregulation of reactive oxygen species [102]. Previous research indicates that reduced expression of the TIGAR gene in an obesity mouse model led to similar negative effects of a high-fat diet [103]. However, the aforementioned work focused on the effect of SIRT3 expression in HFD mice on in vitro cells, but Iljas et al., [104] indicated that mice lacking SIRT3 did not show abnormalities in spindle assembly or chromosome alignment. More interestingly, these parameters in HFD-fed mice as above remained unchanged, and elevated ROS levels did not degrade ATP levels in the oocyte. To what extent these results can be extrapolated to human clinical practice and which exact relevance the described mechanisms could have in women needs to be elucidated.

## 7. Fibrosis and ER stress in the aged ovary and their impact on the oocyte

Fibrosis occurs in numerous aging-related diseases and as we grow older, it is known to cause increased rigidity in the heart, lungs, liver, and also ovary upon an excess accumulation of the extracellular matrix [105]. Recent research provided strong evidence that the stiffness of the ovarian tissue seems to be important for follicular development, growth, and ovulation in advanced-age mothers [106]. As a logical consequence, it appears that the older the mother the lower the chances for an adequate follicular expansion during maturation due to the restricted softness of the ovarian tissue, and the lower the chances that the follicle ruptures at ovulation. However, the molecular and cellular pathways that are responsible for ovarian fibrosis are still not widely investigated. Another important factor that prompted the interest of aging researchers and reproductive medicine is ER stress. The latter-mentioned stress leads to an accumulation of unfolded or misfolded proteins in the ER [107], and in consequence, results in the activation of a specific signal transduction cascade, which is also known as the unfolded protein response (UPR) [107]. Noteworthy, ER stress is activated in oocytes, granulosa cells of growing follicles, and in pre-implantation embryos [107]. ER stress seems to be also relevant to the pathogenesis of the polycystic ovarian syndrome (PCOS) [108-110].

**Figure 4.**
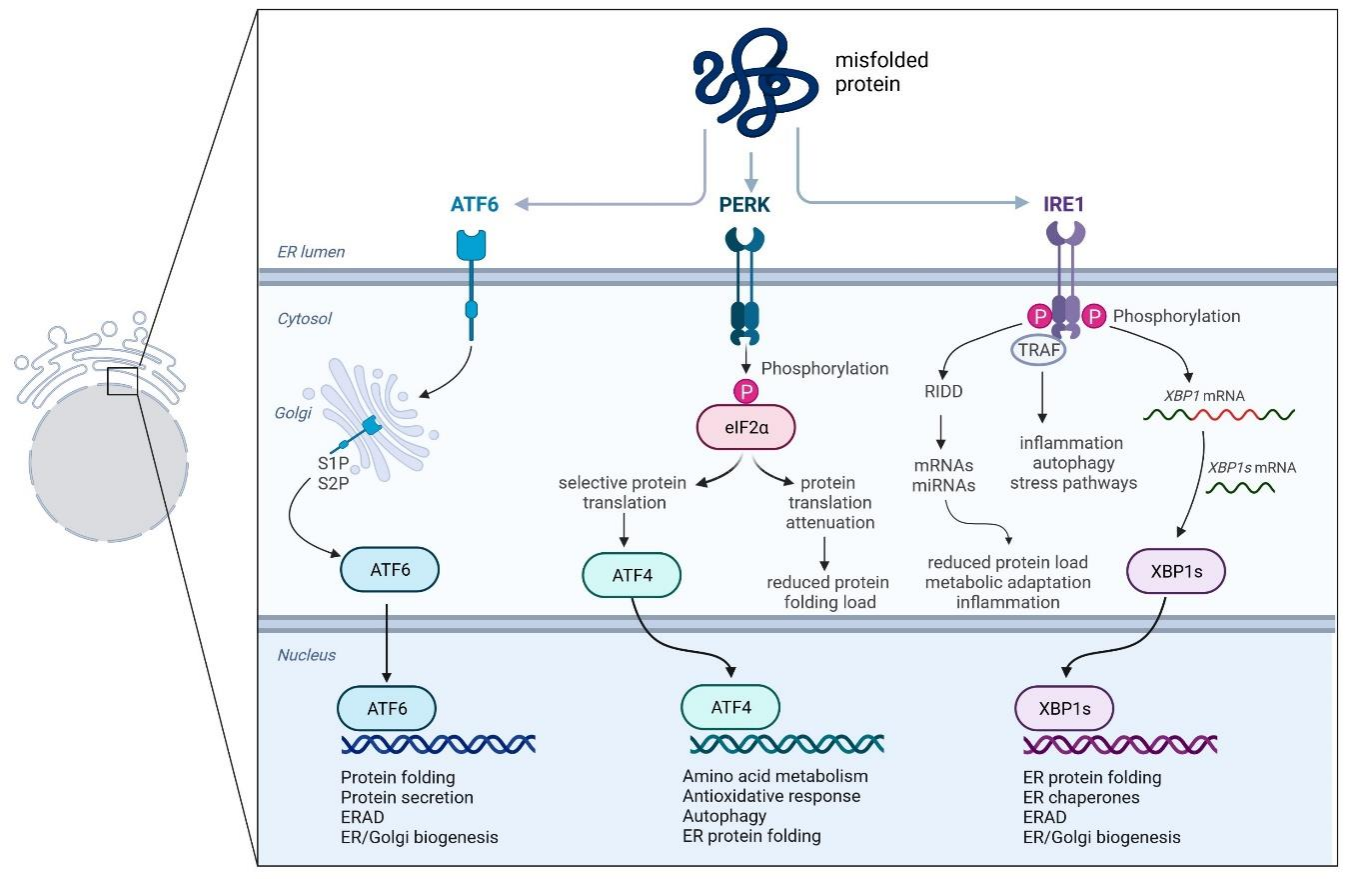
**Scheme showing the three pathways of unfolded protein response (UPR) during ER stress**. The branches of the UPR are characterized by: (left branch) ATF6 passes from the lumen of the ER into the cytosol in the Golgi apparatus, where it is cleaved by site 1 protease (S1P) and site protease 2 (S2P). The cytosolically active fragment of ATF6 migrates to the cell nucleus and activates transcription of UPR target genes involved in ER protein folding homeostasis; (middle branch) PERK phosphorylates the α-subunit of eukaryotic translation initiation factor 2 (eIF2α). Some selective mRNAs, for example, ATF4 mRNA, are preferentially translated when eIF2α is phosphorylated; (right branch) RNase IRE1α is able to cleave ER-bound mRNA or miRNA upon degradation via IRE1-dependent regulated cleavage (RIDD), affecting protein folding charge, cellular metabolism, and inflammatory signaling pathways. The cytosolic domain ofIRE1α may also provide a scaffold for the recruitment of adapter proteins, such as members of the tumor necrosis factor receptor-associated factor (TRAF) family. In addition, IRE1α RNase combines mRNA from XBP1, which encodes a potent transcription factor that activates the expression of UPR target genes involved in ER proteostasis and cellular pathophysiology. Notably, unfolded or misfolded proteins that accumulate in the lumen of the ER can be degraded by proteasome-based ER-associated protein degradation (ERAD).

The folding of secretory proteins is mainly processed by a crucial organelle, the endoplasmic reticulum. Once the latter-mentioned process is disturbed, upon for instance inflammation or oxidative stress, and unfolded or misfolded proteins are accumulated, the so-called ER stress takes place [111-113]. The UPR that is activated during ER stress, consists of three branches: the double-stranded RNA-activated protein kinase-like ER kinase (PERK), the inositol-requiring enzyme 1 (IRE1), and the activating transcription factor 6 (ATF6) [114] (Fig.1). In general, the pathway of UPR aims to correct imbalances of homeostasis and supports in a certain way the survival of cells by three essential processes: 1) attenuation of protein translation, 2) ER chaperones synthesis activation, and 3) ER-associated degradation (ERAD) factors are recruited [107] (Fig. 4). Though the activation of UPR is not always successful to reduce or eliminate ER stress, the pathway of programmed cell death is induced. It is known that activated ER stress induces apoptosis in granulosa cells and cumulus-oocyte-complexes what diminishes the egg’s developmental competence and fertilization chances. Furthermore, ER stress influences steroidogenesis, and therefore, reduces follicular health and luteal function [107]. Interestingly, a moderate degree of ER stress in granulosa- or cumulus cells appears to be beneficial for oocyte maturation [107]. Noteworthy, previous studies provided strong evidence that in granulosa cells of advanced-age women there is an accumulation of so- called advanced glycation end products (AGE). In consequence, an increase of AGE in ovarian follicles of aged women appears to decrease the developmental competence of the egg by triggering inflammation in the surrounding microenvironment [115]. Although it is known that ER stress is linked to the elevation of pro-inflammatory and profibrotic cytokines in granulosa cells [115,116], it is still elusive if and how ER stress is responsible for changes in the ovarian stroma during maternal aging [117-119]. Per se, fibrosis can be characterized as a pathological state or mechanism upon an aberrant inflammatory injury, or an enormous accumulation of fibrous connective tissue occurring in numerous human organs while growing old. Moreover, excessive or progressive fibrosis might lead to severe tissue architecture destruction and even organ failure [105]. Interestingly, an elevated collagen accumulation reflecting symptoms of aging-related stromal fibrosis has been shown both in human, post-menopausal ovaries [118,120] as well as in reproductively aged animals [117,118,121]. Although in other organs the cause of fibrosis is well described, the pathways leading to ovarian fibrosis remain in some parts elusive. Furthermore, the question if there is a correlation between the aging- related fertility drop and changing ovarian architecture needs further elucidation. It appears likely that a chronic accumulation of collagen in the ovarian microenvironment with advancing maternal age restricts a proper ovulation process. There is a clear consensus about the fact that a physiologic ovulation process necessitates the remodeling of the extracellular matrix in such a way that the ruptured ovarian follicle releases adequately the oocyte to the fallopian tube. Therefore, there is no doubt that excessive collagen in the ovary- in other words, a high tissue stiffness, works like a barrier. Interestingly, fibrosis has been also characterized as a pre-metastatic niche for cancer metastasis in models for lung fibrosis, and fibrosis in the mammary gland [122,123]. However, the reason why these metastases are attached to the ovary remains unknown. How the level of fibrosis in the ovary can be diminished will be discussed in the following section.

## 8. ER stress and fibrosis as therapeutic targets to extend ovarian lifespan

So far, anti-fibrotic compounds such as pirfenidone and nintedanib have been approved and used for the treatment of pulmonary diseases, to slow down the fibrosis progression [124,125]. Previous studies showed that (O-[3-piperidino-2-hydroxy-1-propyl]-nicotinic amidoxime) BGP-15 improves the mitochondrial function in several disease models [126,127]. Besides, metformin appears to reduce fibrosis in different pre-clinical studies via the AMPK-mediated pathway [128,129]. Interestingly, in a very recent mouse model, it has been shown that both anti-fibrosis compounds pirfenidone and BGP-15 are able to reduce the level of fibrotic collagen, and restored ovulation rate in aged females when compared to untreated counterparts [106]. The BGP-15 treatment did also elevate the levels of extracellular matrix remodeling enzymes such as matrix metalloproteinase Mmp13, indicating its possible role in the process of collagen removal [106]. Moreover, in the same study, authors have shown that BGP-15 reverses mitochondrial dysfunction, oxidative damage, and ER stress in aged mice what in consequence, has also positive effects on the oocyte [106]. The use of metformin has been shown to prevent ovarian fibrosis related to maternal aging [120]. Interestingly, in the latter-mentioned study, the authors postulate that ovarian fibrosis seems to correlate with immune and stromal properties which are known as a tumor-permissive niche [120]. As a logical consequence, these findings could help to answer the question of why metastases of other primary tumors are localized in the ovary. Noteworthy, probiotics also appear to have a positive effect when used to treat cystic fibrosis, and reduce ER stress, too [130-132]. In conclusion, above-mentioned compounds have a certain potential to extend the ovarian life span via the modulation of ER stress and fibrotic collagen.

## 9. Future research directions and conclusions

Novel research data raised the question of whether the modulation of epigenetic reprogramming, for instance with the help of gene therapy (or DNA methylation editing), could support tissue repair in the ovary and could therefore enable a partial reversal of the aging-related quality decline in oocytes. Interestingly, in a recent study

in mice, researchers have postulated that a reduction of ovulation cycles minimizes the risk of oocyte aneuploidy in advanced-age mothers; in other words, keeps their oocytes young [133]. Aberrant chromosome segregation, such as the before-mentioned aneuploidy, can lead to miscarriage or birth defects. The elucidation of novel pathways crucial for mammalian oocyte spindle assembly, particularly in eggs from donors of advanced reproductive age, could lead to the development of adequate medical interventions. Noteworthy, a high degree of aneuploidy in human oocytes is a major cause of pregnancy loss and infertility in women [134]. Especially, defects in spindle assembly have been associated with high levels of aneuploidy in human eggs [135]. However, the mechanisms that facilitate spindle assembly in human oocytes remain elusive. Spindle microtubule nucleation after nuclear envelope disintegration (NEBD) is significantly delayed in human oocytes compared to mitotic cells, and human oocyte chromosomes undergo rapid movement after NEBD and before spindle assembly begins [135]. Moreover, it has been reported that an error-prone chromosome-mediated spindle assembly favors chromosome segregation defects in oocytes of women [136]. Worth mentioning, previous research provided strong evidence that the possibility of aneuploidy increases by more than 50% in parallel to increasing maternal age both in human and mouse oocytes [137]. With regard to the process of chromosome segregation, it is important to know that kinesins hydrolyze approximately 125 ATP molecules for each microtubule binding event [13]. This clearly demonstrates the enormous need for a continuous high energy supply in the oocyte during the spindle assembly which is provided by mitochondrial oxidative phosphorylation (OXPHOS) [13]. However, the latter-mentioned supply of ATP by mitochondria decreases with advancing maternal age [13], and it has been shown that oxidative stress was accompanied by a significant reduction in mitochondrial ATP production and oocyte spindle assembly impairment [138]. Interestingly, the entire meiotic apparatus appears to be negatively affected by decreased mitochondrial potential (ΔΨm) levels such as during mitochondrial dysfunction [139]. Using in vitro models of murine oocyte maturation, metformin has been reported to improve chromosomal segregation accuracy once oxidative stress and mitochondrial dysfunction were reduced upon treatment, [140]. In recent years, various mechanisms have been described that play a role in age-related maternal aneuploidy, which has been reviewed elsewhere [141].

Interestingly, the production line theory states that the best oocytes are released from follicles first at relatively young reproductive ages, while those with diminished quality are ovulated at a more advanced age. Lowering the frequency of ovulations and protecting youthful epigenetic marks could represent a novel strategy to postpone the effects of aging upon oocytes. This gives hope of future interventions to counteract the negative effects of epigenetic aging in oocytes, specifically in IVF of reproductively aged patients. In conclusion, the power of epigenetics did lay a cornerstone for a new era of research and medicine and opened new possibilities to decipher the molecular codes of oocyte longevity. The constantly increasing number of people with associated metabolic diseases is a difficult challenge for clinicians. Maternal age, DNA methylation, and obesity are factors, which have a significant impact on female mammalian gamete and embryonic development. Importantly though, attention should be paid to genes and proteins involved in the regulation of epigenetic mechanisms and the oocyte’s development including Stella, TET3, UHRF1, SIRT3 as well as m6a, and components of its complex. As it turns out, disorders related to the functioning of the aforementioned structures lead to impaired oogenesis, early embryonic development, and epigenetic reprogramming mechanisms in the embryo. Taken together, elucidating the molecular mechanisms which take place in the oocytes under the influence of aging, obesity, and DNA methylation will help to implement adequate interventions which are able to reduce the negative effects of the before-mentioned factors. Furthermore, in our Review, we aimed to connect the recent findings of these factors, which are mainly made in animal models, and emphasize their relevance for the mammalian egg. To what extent these findings are translational for the human egg needs further elucidation. However, the animal results could be a first hint to identify therapeutic targets to improve oocyte health. Therefore, this commentary aimed to stimulate new interesting research projects to decipher the code of the oocyte’s health- and life span. The above-mentioned effects of pirfenidone, BGP-15, and other substances in animal models of ovarian aging have to be treated with caution since the used concentrations are often very high compared to the physiological levels. Compounds, like for instance senolytics, that are targeting not only aging-related fibrosis, but also mitochondrial dysfunction could be an interesting therapeutic strategy to maintain ovarian function in women with premature ovarian aging or metabolic disorders. Noteworthy, the use of senolytics in different trials or models for aging-related pathologies needs further elucidation, before these compounds could be safely used to prevent, delay or reverse the aging phenotype and related diseases. However, research on senolytics is rapidly advancing, and rigorous pre-clinical and translational experiments, also with regard to the ovary, should be performed. Since the birth of the world’s first IVF baby born in 1978, there is still a big need to assess the long-term consequences of assisted reproductive technologies, particularly on age-related phenotypes, in children conceived with the help of these techniques. Especially, the two cornerstones such as excessive body weight and aging are common symptoms in infertile or subfertile women. This still leads to a dilemma for doctors and specialists in human reproductive medicine of whether to encourage weight loss first or to start infertility treatment immediately. Despite their well-known effects on fertility, studies evaluating the combined effect of women's age and body mass index (BMI) on cumulative live birth rates are still rare and conflicting. According to a very recent study, an intervention to induce weight loss in women prior to ART appears to be beneficial, particularly in women younger than 35 years with a BMI ≥ 25 kg/m^2^. Therefore, for clinical practice, an individualized strategy that takes into account maternal age, patient’s weight, diet, and lifestyle may be the best course of action [142].

## References

[1] Mastenbroek S, de Wert G, Adashi EY (2021). The Imperative of Responsible Innovation in Reproductive Medicine. N Engl J Med, 385(22):2096-2100. 34818487 10.1056/NEJMsb2101718

[2] Kemkes-Grottenthaler A (2003). Postponing or rejecting parenthood? Results of a survey among female academic professionals. J Biosoc Sci, 35:213-226. 12664959 10.1017/s002193200300213x

[3] Seals DR, Justice JN, Larocca TJ (2016). Physiological geroscience: targeting function to increase healthspan and achieve optimal longevity. J Physiol, 594:2001-2024. 25639909 10.1113/jphysiol.2014.282665 PMC4933122

[4] Gleicher N, Barad DH, Adashi EY (2020). Why is use of donor eggs not viewed as treatment failure? A call for improvements in treatments with autologous oocytes. J Assist Reprod Genet, 37:1583-1588. 32504304 10.1007/s10815-020-01847-x PMC7376996

[5] López-Otín C, Blasco MA, Partridge L, Serrano M, Kroemer G (2013). The hallmarks of aging. Cell, 153:1194-217. 23746838 10.1016/j.cell.2013.05.039 PMC3836174

[6] Arneson A, Haghani A, Thompson MJ, Pellegrini M, Kwon SB, Vu H, et al. (2022). A mammalian methylation array for profiling methylation levels at conserved sequences. Nat Commun, 13:783. 35145108 10.1038/s41467-022-28355-z PMC8831611

[7] Horvath S, Haghani A, Macoretta N, Ablaeva J, Zoller JA, Li CZ, et al. (2022). DNA methylation clocks tick in naked mole rats but queens age more slowly than non breeders. Nat Aging, 2:46-59. 35368774 10.1038/s43587-021-00152-1 PMC8975251

[8] Looker AC, Pfeiffer CM, Lacher DA, Schleicher RL, Picciano MF, Yetley EA (2008). Serum 25-hydroxyvitamin D status of the US population: 1988-1994 compared with 2000-2004. Am J Clin Nutr, 88:1519-1527. 19064511 10.3945/ajcn.2008.26182 PMC2745830

[9] Trasino SE, Tang XH, Jessurun J, Gudas LJ (2015). Obesity leads to tissue, but not serum vitamin A deficiency. Sci Rep, 5:1-10. 10.1038/srep15893 PMC4629132 26522079

[10] Broughton DE, Moley KH (2017). Obesity and female infertility: potential mediators of obesity's impact. Fertil Steril, 107:840-847. 28292619 10.1016/j.fertnstert.2017.01.017

[11] Yang PK, Chou CH, Huang CC, Wen WF, Chen HF, Shun CT, et al. (2021). Obesity alters ovarian follic-ulogenesis through disrupted angiogenesis from increased IL-10 production. Mol Metab, 49:101189. 33592337 10.1016/j.molmet.2021.101189 PMC7933796

[12] Neuber E, Powers RD (2000). Is the mouse a clinically relevant model for human fertilization failures? Hum Reprod, 15:171-174. 10611208 10.1093/humrep/15.1.171

[13] van der Reest J, Cecchino GN, Haigis MC, Kordowitzki P (2021). Mitochondria: Their relevance during oocyte ageing. Ageing Res Rev, 70:101378. 34091076 10.1016/j.arr.2021.101378

[14] Hattori H, Hiura H, Kobayashi N, Takahashi S, Okae H, Arima T (2018). Therapeutic Approaches to Imprinting Diseases. In: Trygve OT editor, Epigenetics in Human Disease, Cambridge: Academic Press, 861-875.

[15] Bostick M, Kim JK, Estève PO, Clark A, Pradhan S, Jacobsen SE (2007). UHRF1 plays a role in maintaining DNA methylation in mammalian cells. Science, 317:1760-1764. 17673620 10.1126/science.1147939

[16] Nakamura T, Arai Y, Umehara H, Masuhara M, Kimura T, Taniguchi H, et al. (2007). PGC7/Stella protects against DNA demethylation in early embryogenesis. Nat Cell Biol, 9:64-71. 17143267 10.1038/ncb1519

[17] Wang QQ, Zhang YM, Zhong X, Li JW, An XR, Hou J (2019). Dimethylated histone H3 lysine 9 is dispensable for the interaction between developmental pluripotency-associated protein 3 (Dppa3) and ten-eleven translocation 3 (Tet3) in somatic cells. Reprod Fertil Dev, 31(2):347-356. 30099980 10.1071/RD18062

[18] Huang J, Ru G, Sun J, Sun L, Li Z (2022). Elevated RIF1 participates in the epigenetic abnormalities of zygotes by regulating histone modifications on MuERV-L in obese mice. Mol Med, 28:1-15. 35123389 10.1186/s10020-022-00446-z PMC8818203

[19] Wasserzug-Pash P, Rothman R, Reich E, Zecharyahu L, Schonberger O, Weiss Y, et al. (2022). Loss of heterochromatin and retrotransposon silencing as determinants in oocyte aging. Aging Cell, 21:e13568. 35166017 10.1111/acel.13568 PMC8920445

[20] Kordowitzki P, Haghani A, Zoller JA, Li CZ, Raj K, Spangler ML, et al. (2021). Epigenetic clock and methylation study of oocytes from a bovine model of reproductive aging. Aging Cell, 2:e13349. 10.1111/acel.13349 PMC8135012 33797841

[21] Li B, Carey M, Workman JL (2007). The role of chromatin during transcription. Cell, 128:707-19. 17320508 10.1016/j.cell.2007.01.015

[22] Wasserzug Pash P, Karavani G, Reich E, Zecharyahu L, Kay Z, Bauman D, et al. (2023). Pre-pubertal oocytes harbor altered histone modifications and chromatin configuration. Front Cell Dev Biol, 10:1060440. 36704200 10.3389/fcell.2022.1060440 PMC9871384

[23] Wasserzug-Pash P, Klutstein M (2019). Epigenetic changes in mammalian gametes throughout their lifetime: the four seasons metaphor. Chromosoma, 128:423-441. 31030260 10.1007/s00412-019-00704-w

[24] Sankar A, Lerdrup M, Manaf A, Johansen JV, Gonzalez JM, Borup R, et al. (2020). KDM4A regulates the maternal-to-zygotic transition by protecting broad H3K4me3 domains from H3K9me3 invasion in oocytes. Nat Cell Biol, 22:380-388. 32231309 10.1038/s41556-020-0494-z PMC7212036

[25] Tiku V, Antebi A (2018). Nucleolar Function in Lifespan Regulation. Trends Cell Biol, 28:662-672. 29779866 10.1016/j.tcb.2018.03.007

[26] Wang M, Lemos B (2019). Ribosomal DNA harbors an evolutionarily conserved clock of biological aging. Genome Res, 29:325-333. 30765617 10.1101/gr.241745.118 PMC6396418

[27] Potabattula R, Trapphoff T, Dittrich M, Fic K, Ptak GE, Dieterle S, et al. (2022). Ribosomal DNA methylation in human and mouse oocytes increases with age. Aging, 14:1214-1232. 35157611 10.18632/aging.203891 PMC8876901

[28] Cimadomo D, Fabozzi G, Vaiarelli A, Ubaldi N, Ubaldi FM, Rienzi L (2018). Impact of Maternal Age on Oocyte and Embryo Competence. Front Endocrinol, 29:9:327. 10.3389/fendo.2018.00327 PMC6033961 30008696

[29] Ge ZJ, Schatten H, Zhang CL, Sun QY (2015). Oocyte ageing and epigenetics. Reproduction, 149:R103-14. 25391845 10.1530/REP-14-0242 PMC4397590

[30] Castillo-Fernandez J, Herrera-Puerta E, Demond H, Clark SJ, Hanna CW, Hemberger M, et al. (2020). Increased transcriptome variation and localised DNA methylation changes in oocytes from aged mice revealed by parallel single-cell analysis. Aging Cell, 19:e13278. 33201571 10.1111/acel.13278 PMC7744954

[31] Booth LN, Brunet A (2016). The Aging Epigenome. Mol Cell, 62:728-44. 27259204 10.1016/j.molcel.2016.05.013 PMC4917370

[32] Bell CG, Lowe R, Adams PD, Baccarelli AA, Beck S, Bell JT, Christensen BC, Gladyshev VN, Heijmans BT, Horvath S, Ideker T, Issa JJ, Kelsey KT, Marioni RE, Reik W, Relton CL, Schalkwyk LC, Teschendorff AE, Wagner W, Zhang K, Rakyan VK (2019). DNA methylation aging clocks: challenges and recommendations. Genome Biol, 20:249. 31767039 10.1186/s13059-019-1824-y PMC6876109

[33] Horvath S, Raj K (2018). DNA methylation-based biomarkers and the epigenetic clock theory of ageing. Nat Rev Genet, 19:371-384. 29643443 10.1038/s41576-018-0004-3

[34] Arbeithuber B, Hester J, Cremona MA, Stoler N, Zaidi A, Higgins B, et al. (2020). Age-related accumulation of de novo mitochondrial mutations in mammalian oocytes and somatic tissues. PLoS Biol, 18:e3000745. 32667908 10.1371/journal.pbio.3000745 PMC7363077

[35] Kerepesi C, Zhang B, Lee SG, Trapp A, Gladyshev VN (2021). Epigenetic clocks reveal a rejuvenation event during embryogenesis followed by aging. Sci Adv, 7:eabg6082. 34172448 10.1126/sciadv.abg6082 PMC8232908

[36] Saenz-de-Juano MD, Ivanova E, Billooye K, Herta AC, Smitz J, Kelsey G, et al. (2019). Genome-wide assessment of DNA methylation in mouse oocytes reveals effects associated with in vitro growth, superovulation, and sexual maturity. Clin Epigenetics, 11:1-19. 31856890 10.1186/s13148-019-0794-y PMC6923880

[37] Sendžikaitė G, Kelsey G. (2019). The role and mechanisms of DNA methylation in the oocyte. Essays Biochem, 63:691-705 31782490 10.1042/EBC20190043 PMC6923320

[38] Tucci V, Isles AR, Kelsey G, Ferguson-Smith AC, Bartolomei MS, Benvenisty et al. (2019). Genomic imprinting and physiological processes in mammals. Cell, 176(5):952-965. 30794780 10.1016/j.cell.2019.01.043

[39] Li Y, Li J (2019). Technical advances contribute to the study of genomic imprinting. PLoS genetics, 15:e1008151. 31220079 10.1371/journal.pgen.1008151 PMC6586256

[40] Lyko F (2018). The DNA methyltransferase family: a versatile toolkit for epigenetic regulation. Nature Reviews Genetics, 19(2):81-92. 10.1038/nrg.2017.80 29033456

[41] Aapola U, Shibuya K, Scott HS, Ollila J, Vihinen M, Heino M, et al. (2000). Isolation and initial characterization of a novel zinc finger gene, DNMT3L, on 21q22. 3, related to the cytosine-5-methyltransferase 3 gene family. Genomics, 65(3):293-298. 10857753 10.1006/geno.2000.6168

[42] Jia D, Jurkowska RZ, Zhang X, Jeltsch A, Cheng X (2007). Structure of Dnmt3a bound to Dnmt3L suggests a model for de novo DNA methylation. Nature, 449(7159):248-251 17713477 10.1038/nature06146 PMC2712830

[43] Kaneda M, Hirasawa R, Chiba H, Okano M, Li E, Sasaki H (2010). Genetic evidence for Dnmt3a-dependent imprinting during oocyte growth obtained by conditional knockout with Zp3-Cre and complete exclusion of Dnmt3b by chimera formation. Genes to Cells, 15(3):169-179. 20132320 10.1111/j.1365-2443.2009.01374.x

[44] Barau J, Teissandier A, Zamudio N, Roy S, Nalesso V, Hérault Y, et al. (2016). The DNA methyltransferase DNMT3C protects male germ cells from transposon activity. Science, 354(6314):909-912. 27856912 10.1126/science.aah5143

[45] Jain D, Meydan C, Lange J, Claeys Bouuaert C, Lailler N, Mason CE, et al. (2017). rahu is a mutant allele of Dnmt3c, encoding a DNA methyltransferase homolog required for meiosis and transposon repression in the mouse male germline. PLoS genetics, 13(8):e1006964. 28854222 10.1371/journal.pgen.1006964 PMC5607212

[46] Lucifero D, La Salle S, Bourc'his D, Martel J, Bestor TH, Trasler JM (2007). Coordinate regulation of DNA methyltransferase expression during oogenesis. BMC developmental biology, 7:1-14. 17445268 10.1186/1471-213X-7-36 PMC1878483

[47] Du W, Dong Q, Zhang Z, Liu B, Zhou T, Xu RM, et al. (2019). Stella protein facilitates DNA demethylation by disrupting the chromatin association of the RING finger-type E3 ubiquitin ligase UHRF1. J Biol Chem, 294:8907-8917. 31018966 10.1074/jbc.RA119.008008 PMC6552428

[48] Han L, Ren C, Zhang J, Shu W, Wang Q (2019). Differential roles of Stella in the modulation of DNA methylation during oocyte and zygotic development. Cell Disc, 5:1-4. 10.1038/s41421-019-0081-2 PMC6349861 30701082

[49] Gu TP, Guo F, Yang H, Wu HP, Xu GF, Liu W, et al. (2011). The role of Tet3 DNA dioxygenase in epigenetic reprogramming by oocytes. Nature, 477:606-610. 21892189 10.1038/nature10443

[50] Iqbal K, Jin SG, Pfeifer GP, Szabó PE (2011). Reprogramming of the paternal genome upon fertilization involves genome-wide oxidation of 5-methylcytosine. Proc Natl Acad Sci, 108:3642-3647. 21321204 10.1073/pnas.1014033108 PMC3048122

[51] Nakamura T, Liu YJ, Nakashima H, Umehara H, Inoue K, Matoba S, et al. (2012). PGC7 binds histone H3K9me2 to protect against conversion of 5mC to 5hmC in early embryos. Nature, 486:415-419. 22722204 10.1038/nature11093

[52] Zeng Y; Chen T (2019). DNA methylation reprogramming during mammalian development. Genes, 10:257. 30934924 10.3390/genes10040257 PMC6523607

[53] Wu J, Huang BO, Chen HE, Yin Q, Liu Y, Xiang Y, et al. (2016). The landscape of accessible chromatin in mammalian preimplantation embryos. Nature, 534:652-657. 27309802 10.1038/nature18606

[54] Huang Y, Kim JK, Do DV, Lee C, Penfold CA, Zylicz JJ, et al. (2017). Stella modulates transcriptional and endogenous retrovirus programs during maternal-to-zygotic transition. Elife, 6:e22345. 28323615 10.7554/eLife.22345 PMC5404928

[55] Han L, Ren C, Li L, Li X, Ge J, Wang H, (2018). Embryonic defects induced by maternal obesity in mice derive from Stella insufficiency in oocytes. Nat Gen, 50:432-442. 10.1038/s41588-018-0055-6 29459681

[56] Arand J, Chiang HR, Martin D Snyder MP, Sage J, Reijo Pera RA, et al. (2022). Tet enzymes are essential for early embryogenesis and completion of embryonic genome activation. EMBO Rep, 23:e53968. 34866320 10.15252/embr.202153968 PMC8811641

[57] Mark W, Julia A, Vittorio S, Konstantin L, Michele B, Richard R, et al. (2014). Dynamic link of DNA demethylation, DNA strand breaks and repair in mouse zygotes. EMBO J, 29:1877-1888. 10.1038/emboj.2010.80 PMC2885932 20442707

[58] Li Y, Zhang Z, Chen J, Liu W, Lai W, Liu B, et al. (2018). Stella safeguards the oocyte methylome by preventing de novo methylation mediated by DNMT1. Nature, 564:136-140. 30487604 10.1038/s41586-018-0751-5

[59] Arand J, Spieler D, Karius T, Branco MR, Meilinger D, Meissner A, et al. (2012). In vivo control of CpG and non-CpG DNA methylation by DNA methyltransferases. PLoS Genet, 8:e1002750. 22761581 10.1371/journal.pgen.1002750 PMC3386304

[60] Shin SW, Vogt EJ, Jimenez-Movilla M, Baibakov B, Dean J (2017). Cytoplasmic cleavage of DPPA3 is required for intracellular trafficking and cleavage-stage development in mice. Nat Commun, 8:1643. 29158485 10.1038/s41467-017-01387-6 PMC5696369

[61] Maenohara S, Unoki M, Toh H, Ohishi H, Sharif J, Koseki H, et al. (2017). Role of UHRF1 in de novo DNA methylation in oocytes and maintenance methylation in preimplantation embryos. PLoS genetics, 13:e1007042. 28976982 10.1371/journal.pgen.1007042 PMC5643148

[62] Roundtree IA, Evans ME, Pan T, He C (2017). Dynamic RNA modifications in gene expression regulation. Cell, 169:1187-1200. 28622506 10.1016/j.cell.2017.05.045 PMC5657247

[63] Heck AM, Wilusz CJ (2019). Small changes, big implications: the impact of m6A RNA methylation on gene expression in pluripotency and development. Biochim Biophys Acta Gene Regul Mech BBA-Gene Regul Mech, 1862:194402. 10.1016/j.bbagrm.2019.07.003 PMC6742438 31325527

[64] Kan L, Grozhik AV, Vedanayagam J, Patil DP, Pang N, Lim KS, et al. (2017). The m6A pathway facilitates sex determination in Drosophila. Nat Commun, 8:1-16. 28675155 10.1038/ncomms15737 PMC5500889

[65] Ping XL, Sun BF, Wang L, Xiao W, Yang X, Wang WJ, et al. (2014). Mammalian WTAP is a regulatory subunit of the RNA N6-methyladenosine methyltransferase. Cell Res, 24:177-189. 24407421 10.1038/cr.2014.3 PMC3915904

[66] Yue Y, Liu J, Cui X, Cao J, Luo G, Zhang Z, et al. (2018). VIRMA mediates preferential m6A mRNA methylation in 3′ UTR and near stop codon and associates with alternative polyadenylation. Cell Discov, 4:1-17. 29507755 10.1038/s41421-018-0019-0 PMC5826926

[67] Patil DP, Chen CK, Pickering BF, Chow A, Jackson C, Guttman M, et al. (2016). m6A RNA methylation promotes XIST-mediated transcriptional repression. Nature, 537:369-373. 27602518 10.1038/nature19342 PMC5509218

[68] Knuckles P, Lence T, Haussmann IU, Jacob D, Kreim N, Carl SH, et al. (2018). Zc3h13/Flacc is required for adenosine methylation by bridging the mRNA-binding factor Rbm15/Spenito to the m6A machinery component Wtap/Fl (2) d. Gen Develop, 32:415-429. 10.1101/gad.309146.117 PMC5900714 29535189

[69] Wen J, Lv R, Ma H, Shen H, He C, Wang J, et al. (2018). Zc3h13 regulates nuclear RNA m6A methylation and mouse embryonic stem cell self-renewal. Mol Cell, 69:1028-1038. 29547716 10.1016/j.molcel.2018.02.015 PMC5858226

[70] Pendleton KE, Chen B, Liu K, Hunter OV, Xie Y, Tu B, et al. (2017). The U6 snRNA m6A methyltransferase METTL16 regulates SAM synthetase intron retention. Cell, 169:824-835. 28525753 10.1016/j.cell.2017.05.003 PMC5502809

[71] Yang Y, Hsu PJ, Chen YS, Yang YG (2018). Dynamic transcriptomic m6A decoration: writers, erasers, readers and functions in RNA metabolism. Cell Res, 28:616-624. 29789545 10.1038/s41422-018-0040-8 PMC5993786

[72] Jia G, Fu YE, Zhao XU, Dai Q, Zheng G, Yang Y, et al. (2011). N6-methyladenosine in nuclear RNA is a major substrate of the obesity-associated FTO. Nat Chem Biol, 7:885-887. 22002720 10.1038/nchembio.687 PMC3218240

[73] Zheng G, Dahl JA, Niu Y, Fedorcsak P, Huang CM, Li CJ, et al. (2013). ALKBH5 is a mammalian RNA demethylase that impacts RNA metabolism and mouse fertility. Mol Cell, 49:18-29. 23177736 10.1016/j.molcel.2012.10.015 PMC3646334

[74] Tang C, Klukovich R, Peng H, Wang Z, Yu T, Zhang Y, et al. (2018). ALKBH5-dependent m6A demethylation controls splicing and stability of long 3′-UTR mRNAs in male germ cells. Proc Natl Acad Sci, 115:325-333. 29279410 10.1073/pnas.1717794115 PMC5777073

[75] Shi R, Ying S, Li Y, Zhu L, Wang X, Jin H (2021). Linking the YTH domain to cancer: the importance of YTH family proteins in epigenetics. Cell Death Dis, 12, 1-14. 33795663 10.1038/s41419-021-03625-8 PMC8016981

[76] Huang H, Weng H, Sun W, Qin X, Shi H, Wu H, et al (2018). Recognition of RNA N6-methyladenosine by IGF2BP proteins enhances mRNA stability and translation. Nat Cell Biol, 20, 285-295. 29476152 10.1038/s41556-018-0045-z PMC5826585

[77] Mu H, Zhang T, Yang Y, Zhang D, Gao J, Li J, et al (2021). METTL3-mediated mRNA N6-methyladenosine is required for oocyte and follicle development in mice. Cell Death Dis 2021, 12, 1-13. 10.1038/s41419-021-04272-9 PMC8542036 34689175

[78] Geula S, Moshitch-Moshkovitz S, Dominissini D, Mansour AA, Kol N, Salmon-Divon M, et al (2015). m6A mRNA methylation facilitates resolution of naïve pluripotency toward differentiation. Science, 347, 1002-1006. 25569111 10.1126/science.1261417

[79] Zhang J, Ma R, Li L, Wang L, Hou X, Han L, et al (2017). Intersectin 2 controls actin cap formation and meiotic division in mouse oocytes through the Cdc42 pathway. FASEB J, 31, 4277-4285. 28626024 10.1096/fj.201700179R

[80] Zhang M, Zhai Y, Zhang S, Dai X, Li Z (2020). Roles of N6-Methyladenosine (m6A) in stem cell fate decisions and early embryonic development in mammals. Front Cell Dev Biol, 8, 782. 32850871 10.3389/fcell.2020.00782 PMC7431753

[81] Ma JY, Li MO, Luo YB, Song S, Tian D, Yang J, et al (2013). Maternal factors required for oocyte developmental competence in mice: transcriptome analysis of non-surrounded nucleolus (NSN) and surrounded nucleolus (SN) oocytes. Cell cycle, 12(12), 1928-1938. 23673344 10.4161/cc.24991 PMC3735707

[82] Wu Y, Xu X, Qi M, Chen C, Li M, Yan R, et al (2022). N6-methyladenosine regulates maternal RNA maintenance in oocytes and timely RNA decay during mouse maternal-to-zygotic transition. Nat Cell Biol, 24, 917-927. 35606490 10.1038/s41556-022-00915-x

[83] Sommerkamp P(2022). Substrates of the m6A demethylase FTO: FTO-LINE1 RNA axis regulates chromatin state in mESCs. Signal Transduct Target Ther, 7, 1-3. 35794111 10.1038/s41392-022-01085-w PMC9259694

[84] Merkestein M, Laber S, McMurray F, Andrew D, Sachse G, Sanderson J, et al (2015). FTO influences adipogenesis by regulating mitotic clonal expansion. Nat Comm, 6, 1-9. 10.1038/ncomms7792 PMC4410642 25881961

[85] Wei J, Yu X, Yang L, Liu X, Gao B, Huang B, et al (2022). FTO mediates LINE1 m6A demethylation and chromatin regulation in mESCs and mouse development. Science, 376, 968-973. 35511947 10.1126/science.abe9582 PMC9746489

[86] Jachowicz JW, Bing X, Pontabry J, Bošković A, Rando OJ, Torres-Padilla ME (2017). LINE-1 activation after fertilization regulates global chromatin accessibility in the early mouse embryo. Nat Genet 2017, 49, 1502-1510. 10.1038/ng.3945 28846101

[87] Hu Y, Ouyang Z, Sui X, Qi M, Li M, He Y et al (2020). Oocyte competence is maintained by m6A methyltransferase KAA1429-mediated RNA metabolism during mouse follicular development. Cell Death Differ, 27, 2468-2483. 32094512 10.1038/s41418-020-0516-1 PMC7370231

[88] Mitchell S, Shaw D (2015). The worldwide epidemic of female obesity. Best practice & research Clinical obstetrics & gynaecology, 29(3), 289-299. 25487257 10.1016/j.bpobgyn.2014.10.002

[89] Hensrud DD, Klein S (2006). Extreme obesity: a new medical crisis in the United States. In Mayo Clinic Proceedings, Vol. 81, No. 10, pp. S5-S10. 10.1016/s0025-6196(11)61175-0 17036573

[90] Snider AP, Wood JR (2019). Obesity induces ovarian inflammation and reduces oocyte quality. Reproduction, 158(3), R79-R90. 30999278 10.1530/REP-18-0583

[91] Gonzalez MB, Robker RL, Rose RD (2022). Obesity and oocyte quality: significant implications for ART and emerging mechanistic insights. Biology of Reproduction, 106(2), 338-350. 34918035 10.1093/biolre/ioab228

[92] Silvestris E, de Pergola G, Rosania R, Loverro G (2018). Obesity as disruptor of the female fertility. Reproductive Biology and Endocrinology, 16, 1-13. 29523133 10.1186/s12958-018-0336-z PMC5845358

[93] Ouchi N, Parker JL, Lugus JJ, Walsh K (2011). Adipokines in inflammation and metabolic disease. Nature reviews immunology, 11(2), 85-97. 10.1038/nri2921 PMC3518031 21252989

[94] Nteeba J, Ganesan S, Keating AF (2014). Progressive obesity alters ovarian folliculogenesis with impacts on pro-inflammatory and steroidogenic signaling in female mice. Biology of Reproduction, 91(4), 86-1. 25143355 10.1095/biolreprod.114.121343 PMC4435031

[95] Ge H, Tollner TL, Hu Z, Dai M, Li X, Guan H, et al (2012). The importance of mitochondrial metabolic activity and mitochondrial DNA replication during oocyte maturation in vitro on oocyte quality and subsequent embryo developmental competence. Molecular reproduction and development, 79(6), 392-401. 22467220 10.1002/mrd.22042

[96] Wakai T, Harada Y, Miyado K, Kono T (2014). Mitochondrial dynamics controlled by mitofusins define organelle positioning and movement during mouse oocyte maturation. Molecular human reproduction, 20(11), 1090-1100. 25113836 10.1093/molehr/gau064

[97] Luzzo KM, Wang Q, Purcell SH, Chi M., Jimenez PT, Grindler N, et al (2012). High fat diet induced developmental defects in the mouse: oocyte meiotic aneuploidy and fetal growth retardation/brain defects. PloS one, 7(11), e49217. 23152876 10.1371/journal.pone.0049217 PMC3495769

[98] Tao R, Vassilopoulos A, Parisiadou L, Yan Y, Gius D (2014). Regulation of MnSOD enzymatic activity by Sirt3 connects the mitochondrial acetylome signaling networks to aging and carcinogenesis. Antioxid Redox Signal 2014, 20, 1646-1654 10.1089/ars.2013.5482 PMC3942696 23886445

[99] Qiu X, Brown K, Hirschey MD, Verdin E, Chen D (2010). Calorie restriction reduces oxidative stress by SIRT3-mediated SOD2 activation. Cell Metab, 12, 662-667. 21109198 10.1016/j.cmet.2010.11.015

[100] Zhao HC, Ding T, Ren Y, Li TJ, Li R, Fan Y (2016). Role of Sirt3 in mitochondrial biogenesis and developmental competence of human in vitro matured oocytes. Hum Reprod 2016, 31, 607-622. 10.1093/humrep/dev345 26787646

[101] Han L, Wang H, Li L, Li X, Ge J, Reiter RJ, et al (2017). Melatonin protects against maternal obesity-associated oxidative stress and meiotic defects in oocytes via the SIRT 3-SOD 2-dependent pathway. J Pineal Res, 63, e12431. 10.1111/jpi.12431 28658527

[102] Tang J, Chen L, Qin ZH, Sheng R (2021). Structure, regulation, and biological functions of TIGAR and its role in diseases. Acta Pharmacol Sin, 42, 1547-1555. 33510458 10.1038/s41401-020-00588-y PMC8463536

[103] Wang H, Cheng Q, Li X, Hu F, Han L, Zhang H, et al. (2018). Loss of TIGAR induces oxidative stress and meiotic defects in oocytes from obese mice. Mol Cell Proteomics 2018, 17, 1354-1364. 10.1074/mcp.RA118.000620 PMC6030723 29776966

[104] Iljas JD, Homer HA (2020). Sirt3 is dispensable for oocyte quality and female fertility in lean and obese mice. FASEB J, 34, 6641-6653. 32212196 10.1096/fj.202000153R

[105] Yang Z, Jiang S, Shang J, Jiang Y, Dai Y, Xu B et al (2019). LncRNA: Shedding light on mechanisms and opportunities in fibrosis and aging. Ageing Res Rev, 52:17-31. 30954650 10.1016/j.arr.2019.04.001

[106] Umehara T, Winstanley YE, Andreas E, Morimoto A, Williams EJ, Smith KM (2022). Female reproductive life span is extended by targeted removal of fibrotic collagen from the mouse ovary. Sci Adv, 8(24):eabn4564. 35714185 10.1126/sciadv.abn4564 PMC9205599

[107] Harada M, Takahashi N, Azhary JM, Kunitomi C, Fujii T, Osuga Y (2021). Endoplasmic reticulum stress: a key regulator of the follicular microenvironment in the ovary. Mol Hum Reprod, 27(1):gaaa088. 33543293 10.1093/molehr/gaaa088

[108] Rutkowski DT, Kaufman RJ (2007). That which does not kill me makes me stronger: adapting to chronic ER stress. Trends Biochem Sci, 32(10):469-76. 17920280 10.1016/j.tibs.2007.09.003

[109] Walter P, Ron D (2011). The unfolded protein response: from stress pathway to homeostatic regulation. Science, 334(6059):1081-6. 22116877 10.1126/science.1209038

[110] Hetz C, Zhang K, Kaufman RJ (2020). Mechanisms, regulation and functions of the unfolded protein response. Nat Rev Mol Cell Biol, 21(8):421-438. 32457508 10.1038/s41580-020-0250-z PMC8867924

[111] Hetz C, Axten JM, Patterson JB (2019). Pharmacological targeting of the unfolded protein response for disease intervention. Nat Chem Biol, 15(8):764-775. 31320759 10.1038/s41589-019-0326-2

[112] Karna KK, Shin YS, Choi BR, Kim HK, Park JK (2020). The Role of Endoplasmic Reticulum Stress Response in Male Reproductive Physiology and Pathology: A Review. World J Mens Health, 38(4):484-494. 31385474 10.5534/wjmh.190038 PMC7502313

[113] Rocha M, Apostolova N, Diaz-Rua R, Muntane J, Victor VM (2020). Mitochondria and T2D: Role of Autophagy, ER Stress, and Inflammasome. Trends Endocrinol Metab, 31(10):725-741. 32265079 10.1016/j.tem.2020.03.004

[114] Walter P, Ron D (2011). The unfolded protein response: from stress pathway to homeostatic regulation. Science, 334(6059):1081-6. 22116877 10.1126/science.1209038

[115] Takahashi N, Harada M, Azhary JMK, Kunitomi C, Nose E, Terao H, et al (2019). Accumulation of advanced glycation end products in follicles is associated with poor oocyte developmental competence. Mol Hum Reprod, 25(11):684-694. 31504800 10.1093/molehr/gaz050

[116] Takahashi N, Harada M, Hirota Y, Nose E, Azhary JM, Koike H, et al (2017). Activation of Endoplasmic Reticulum Stress in Granulosa Cells from Patients with Polycystic Ovary Syndrome Contributes to Ovarian Fibrosis. Sci Rep, 7(1):10824. 28883502 10.1038/s41598-017-11252-7 PMC5589802

[117] Briley SM, Jasti S, McCracken JM, Hornick JE, Fegley B, Pritchard MT, et al (2016). Reproductive age-associated fibrosis in the stroma of the mammalian ovary. Reproduction, 152(3):245-260. 27491879 10.1530/REP-16-0129 PMC4979755

[118] Amargant F, Manuel SL, Tu Q, Parkes WS, Rivas F, Zhou LT, et al (2020). Ovarian stiffness increases with age in the mammalian ovary and depends on collagen and hyaluronan matrices. Aging Cell, 19(11):e13259. 33079460 10.1111/acel.13259 PMC7681059

[119] Zhang Z, Schlamp F, Huang L, Clark H, Brayboy L (2020). Inflammaging is associated with shifted macrophage ontogeny and polarization in the aging mouse ovary. Reproduction, 159(3):325-337. 31940276 10.1530/REP-19-0330 PMC7066623

[120] McCloskey CW, Cook DP, Kelly BS, Azzi F, Allen CH, Forsyth A, et al (2020). Metformin Abrogates Age-Associated Ovarian Fibrosis. Clin Cancer Res, 26(3):632-642. 31597663 10.1158/1078-0432.CCR-19-0603

[121] Mara JN, Zhou LT, Larmore M, Johnson B, Ayiku R, Amargant F, et al (2020). Ovulation and ovarian wound healing are impaired with advanced reproductive age. Aging (Albany NY), 12(10):9686-9713. 32407290 10.18632/aging.103237 PMC7288922

[122] Olaso E, Santisteban A, Bidaurrazaga J, Gressner AM, Rosenbaum J, Vidal-Vanaclocha F (1997). Tumor-dependent activation of rodent hepatic stellate cells during experimental melanoma metastasis. Hepatology, 26(3):634-42. 9303493 10.1002/hep.510260315

[123] Jacobs TW, Byrne C, Colditz G, Connolly JL, Schnitt SJ (1999). Radial scars in benign breast-biopsy specimens and the risk of breast cancer. N Engl J Med, 340(6):430-6. 9971867 10.1056/NEJM199902113400604

[124] Saito S, Alkhatib A, Kolls JK, Kondoh Y, Lasky JA (2019). Pharmacotherapy and adjunctive treatment for idiopathic pulmonary fibrosis (IPF). J Thorac Dis, 11(Suppl 14):S1740-S1754. 31632751 10.21037/jtd.2019.04.62 PMC6783717

[125] Spagnolo P, Tzouvelekis A, Bonella F (2018). The Management of Patients With Idiopathic Pulmonary Fibrosis. Front Med (Lausanne), 5:148. 30013972 10.3389/fmed.2018.00148 PMC6036121

[126] Ohlen SB, Russell ML, Brownstein MJ, Lefcort F (2017). BGP-15 prevents the death of neurons in a mouse model of familial dysautonomia. Proc Natl Acad Sci U S A, 114(19):5035-5040. 28439028 10.1073/pnas.1620212114 PMC5441694

[127] Gehrig SM, van der Poel C, Sayer TA, Schertzer JD, Henstridge DC, Church JE et al (2012). Hsp72 preserves muscle function and slows progression of severe muscular dystrophy. Nature, 484(7394):394-8. 22495301 10.1038/nature10980

[128] Ursini F, Grembiale RD, D'Antona L, Gallo E, D'Angelo S, Citraro R et al (2016). Oral Metformin Ameliorates Bleomycin-Induced Skin Fibrosis. J Invest Dermatol, 136(9):1892-1894. 27251791 10.1016/j.jid.2016.05.097

[129] Xiao H, Ma X, Feng W, Fu Y, Lu Z, Xu M et al (2010). Metformin attenuates cardiac fibrosis by inhibiting the TGFbeta1-Smad3 signalling pathway. Cardiovasc Res, 87(3):504-13. 20200042 10.1093/cvr/cvq066

[130] Neri LCL, Taminato M, Silva Filho LVRFD (2019). Systematic Review of Probiotics for Cystic Fibrosis Patients: Moving Forward. J Pediatr Gastroenterol Nutr, 68(3):394-399. 30358738 10.1097/MPG.0000000000002185

[131] Coffey MJ, Garg M, Homaira N, Jaffe A, Ooi CY (2020). Probiotics for people with cystic fibrosis. Cochrane Database Syst Rev, 1(1):CD012949. 31962375 10.1002/14651858.CD012949.pub2 PMC6984633

[132] Ferrari E, Monzani R, Saverio V, Gagliardi M, Pańczyszyn E, Raia V et al (2021). Supplements Reduce ER Stress and Gut Inflammation Associated with Gliadin Intake in a Mouse Model of Gluten Sensitivity. Nutrients, 13(4):1221. 33917155 10.3390/nu13041221 PMC8067866

[133] Chatzidaki EE, Powell S, Dequeker BJH, Gassler J, Silva MCC, Tachibana K (2021). Ovulation suppression protects against chromosomal abnormalities in mouse eggs at advanced 233 maternal age. Curr Biol, 20:S0960-9822(21)00905-2. 10.1016/j.cub.2021.06.076 34314679

[134] Gruhn JR, Zielinska AP, Shukla V, Blanshard R, Capalbo A, Cimadomo D et al (2019). Chromosome errors in human eggs shape natural fertility over reproductive life span. Science. 27;365(6460):1466-1469. 10.1126/science.aav7321 PMC7212007 31604276

[135] Harasimov K, Uraji J, Mönnich EU, Holubcová Z, Elder K, Blayney M, et al (2023). Actin-driven chromosome clustering facilitates fast and complete chromosome capture in mammalian oocytes. Nat Cell Biol, 25(3):439-452. 36732633 10.1038/s41556-022-01082-9 PMC10014578

[136] Holubcová Z, Blayney M, Elder K, Schuh M (2015). Human oocytes. Error-prone chromosome-mediated spindle assembly favors chromosome segregation defects in human oocytes. Science, 348(6239):1143-7. 26045437 10.1126/science.aaa9529 PMC4477045

[137] Handyside AH, Montag M, Magli MC, Repping S, Harper J, Schmutzler A, et al (2016). Multiple meiotic errors caused by predivision of chromatids in women of advanced maternal age undergoing in vitro fertilisation. Eur. J. Hum. Genet, 20:742-747. 10.1038/ejhg.2011.272 PMC3376262 22317970

[138] Zhang X, Wu XQ, Lu S, Guo YL, Ma X (2006). Deficit of mitochondria-derived ATP during oxidative stress impairs mouse MII oocyte spindles. Cell Res, 16(10):841-50. 16983401 10.1038/sj.cr.7310095

[139] Wilding M, De Placido G, De Matteo L, Marino M, Alviggi C, Dale B (2003). Chaotic mosaicism in human preimplantation embryos is correlated with a low mitochondrial membrane potential. Fertil Steril, 79(2):340-6. 12568843 10.1016/s0015-0282(02)04678-2

[140] Li WD, Zang CJ, Yin S, Shen W, Sun QY, Zhao M (2020). Metformin protects against mouse oocyte apoptosis defects induced by arecoline. Cell Prolif, 53(7):e12809. 32557964 10.1111/cpr.12809 PMC7377942

[141] Wasielak-Politowska M, Kordowitzki P (2022). Chromosome Segregation in the Oocyte: What Goes Wrong during Aging. Int [J] Mol Sci. 23(5):2880. 10.3390/ijms23052880 PMC8911062 35270022

[142] Rafael F, Rodrigues MD, Bellver J, Canelas-Pais M, Garrido N, Garcia-Velasco JA, et al (2023). The combined effect of BMI and age on ART outcomes. Hum Reprod, 38(5):886-894. 36928306 10.1093/humrep/dead042

